# Biochemical characterization and chemical validation of *Leishmania* MAP Kinase-3 as a potential drug target

**DOI:** 10.1038/s41598-019-52774-6

**Published:** 2019-11-07

**Authors:** Shweta Raj, Gundappa Saha, Santanu Sasidharan, Vikash Kumar Dubey, Prakash Saudagar

**Affiliations:** 1 Department of Biotechnology, National Institute of Technology-Warangal, Telangana State, 506004 India; 2 School of Biochemical Engineering, Indian Institute of Technology-Banaras Hindu University, Uttar Pradesh, 221005 India; 3 Department of Biosciences and Bioengineering, Indian Institute of Technology Guwahati, Guwahati, Assam 781039 India

**Keywords:** Biochemistry, Chemical biology, Molecular biology

## Abstract

Protozoan parasites of the *Leishmania* genus have evolved unique signaling pathways that can sense various environmental changes and trigger stage differentiation for survival and host infectivity. MAP kinase (MAPK) plays a critical role in various cellular activities like cell differentiation, proliferation, stress regulation, and apoptosis. The *Leishmania donovani* MAPK3 (*Ld*MAPK3) is involved in the regulation of flagella length and hence plays an important role in disease transmission. Here, we reported the gene cloning, protein expression, biochemical characterizations, inhibition studies and cell proliferation assay of *Ld*MAPK3. The recombinant purified *Ld*MAPK3 enzyme obeys the Michaelis-Menten equation with K_m_ and V_max_ of *Ld*MAPK3 was found to be 20.23 nM and 38.77 ± 0.71 nmoles ATP consumed/mg *Ld*MAPK3/min respectively. The maximum kinase activity of *Ld*MAPK3 was recorded at 35 °C and pH 7. The *in-vitro* inhibition studies with two natural inhibitors genistein (GEN) and chrysin (CHY) was evaluated against *Ld*MAPK3. The K_i_ value for GEN and CHY were found to be 3.76 ± 0.28 µM and K_i_ = 8.75 ± 0.11 µM respectively. The IC_50_ value for the compounds, GEN and CHY against *L. donovani* promastigotes were calculated as 9.9 µg/mL and 13 µg/mL respectively. Our study, therefore, reports *Ld*MAPK3 as a new target for therapeutic approach against leishmaniasis.

## Introduction

The protozoan parasites of the genus *Leishmania* are responsible for a group of diseases known as Leishmaniasis. This disease is mainly localized in tropical and temperate geographical regions and infects the immune cells of a human body^1^. Leishmaniasis mainly occurs in three clinical forms ranging from less severe, self-curing, ulcerative skin lesions namely cutaneous Leishmaniasis (CL), disturbed mucosal destruction i.e. Mucocutaneous Leishmaniasis (MCL) to the most severe, lethal form causing visceromegaly known as Visceral Leishmaniasis or kala-azar (VL)^2^. According to the World Health Organization report, this disease mostly affects the poorest people around the globe with an estimation of around 700,000 to 1 million new cases per year^3^. Around 300 million people living in 88 countries are at the risk of infection, causing 20,000–30,000 deaths annually (as per WHO 2019 report). Since leishmaniasis is a poor man’s disease; it is mainly associated with poor sanitation habits, malnutrition, population displacement, deforestation, and urbanization. Till date, there are no effective vaccines available commercially for Leishmaniasis, therefore the treatment completely depends on chemotherapy and controlling its transmission^4^. Chemotherapy includes pentavalent antimonials such as sodium stibogluconate and meglumine antimoniate which are considered as first-line drugs for all forms of Leishmaniasis^5^. Unfortunately, the treatment has gradually become ineffective due to the emerging resistance to pentavalent antimonials, especially in India, where more than 60% of Kala-azar patients were already resistant to first-line therapy^6^. Second-line drugs include amphotericin B, paromomycin, pentamidine, and the first oral drug – miltefosine which is used for both antimony-responding and non-responding patients.

However, various disadvantages like high cost, toxicity and developing resistance to these second-line drugs demands for alternative treatment against leishmaniasis^7^. Another problem is the co-infection of leishmaniasis in HIV patients which is making its treatment more challenging^8^. So, there is an urgent necessity for an alternative effective drug formulation with no or minimum side-effects. Leishmaniasis which is caused by a pathogenic parasite *Leishmania* belongs to the kingdom protozoa. It exists in dimorphic form and completes its lifecycle in two forms depending upon the host. The flagellated form called promastigote is extracellular in nature and found in the gut lumen of sandflies that later gets transmitted to a mammalian host during a blood meal^9^. The non-motile amastigote form is intracellular in nature and is found in the macrophage cells of mammals^10^. The transitional developmental stages are triggered by various environmental changes like temperature (26 °C to 37 °C) and pH (7.4 to 5.5) of the insect and mammalian hosts^11–13^. This transition of the stage plays a very crucial role in the survival of pathogen and disease infectivity. Phosphorylated proteins are pivotal for this stage differentiation. This includes various protein kinases, stress proteins, RNA binding proteins, heat shock proteins, and many cytoskeleton proteins^14^.

The signaling proteins are involved in the intracellular and extracellular signal transduction which leads to the stage differentiation and proliferation of the parasite^15–17^. These signaling proteins play an important role in adapting to the extreme host environments and thus are potential drug targets. Mitogen-Activated Protein (MAP) kinases are a good example of signaling pathways that work in a cascade to phosphorylate other proteins. This cascade starts by phosphorylating MAP kinase kinase kinases (M3Ks), that stimulate MAP kinase kinases (M2Ks), which finally activates MAP kinases (MAPKs) for the regulation of various cellular activities like cell proliferation, differentiation, stress response, infectivity and apoptosis^18,19^. The site of dual phosphorylation is highly conserved throughout eukaryotes and contains the TXY motif within the activation loop of MAPK proteins^20^. The MAP kinase pathway of the *Leishmania* genus is mainly composed of two different families of kinase proteins, the STE family and CMGC family^21^. The STE family of kinases consists of 5 putative MAP kinase kinases and around twenty putative MAP kinase kinase kinases. On the other hand, the CMGC family contains around 17 types of putative MAP kinases. In *Leishmania*, there are around 179 protein kinases identified till date and only fifteen of them are known to be classical MAP kinases^22^.

Leishmanial MAPKs play an important role in the development and intracellular survival of the parasite. Different MAPKs are involved in the survival of parasites in the bloodstream like MAPK1and MAPK2 of *L. Mexicana*^23^. *Lmx*MAPK1 also plays a crucial role in antimony resistance^24^. The *L. mexicana* MAPK3 (*Lmx*MPK3) and MAPK9 (*Lmx*MPK9) are found to be involved in the regulation of flagellar length in promastigotes^23,25–29^, while *Lmx*MPK4, *Lmx*MPK7, and *Lmx*MPK10 were found to play an essential role in inducing stage differentiation. Therefore, all these MAP kinases play an important role in the survival and infectivity of *L. mexicana*^30–32^. Thus, the research of *Leishmania* kinome could help in understanding the system behind the adaption of the parasite in extreme host environments that allows the intracellular and extracellular survival during infection^33^.

The current approaches against Leishmaniasis are mainly based on chemotherapy but due to their disadvantages and side effects, eradication became very difficult. The emerging difficulties in the treatment led to open an alternative path based on natural therapeutic drugs. Several phytochemicals such as flavonoids, isoflavonoids, xanthones, lignans, quinones, terpenoids and alkaloids are known for their anti-protozoan properties^34–40^.

Flavonoids are known for their antioxidant properties, anti-cancerous activities, and several other biological properties. Chrysin (5, 7-dihydroxyflavone) is a natural flavonoid found in the *Passiflora sp*.(Passionflower), *Oroxylum indicium* and honey^41^. Chrysin and its analogs were found to be anti-leishmanial and shown both *in-vitro* and *in-vivo* activities^42,43^. Isoflavone such as Genistein is a tyrosine kinase inhibitor and is involved in the phosphorylation of tyrosine induced by collagen^44^. However, most of the anti-leishmanial chemotherapies induce oxidative stress which leads to the DNA damage and mutagenic conditions, Genistein has been shown to reduce the DNA damage caused by meglumine antimoniate^45^. Therefore, these two phytochemicals could emerge as a new novel therapeutic to minimize genotoxic effects. In this present study, we have targeted MAPK3 of *Leishmania donovani* using *in- vitro* approach and identified chrysin (CHY) and genistein (GEN) as an inhibitor of *Ld*MAPK3 and holds a potential to emerge as a new therapeutics against leishmaniasis.

## Results

### Sequence characterization and analysis of *Ld*MAPK3

**Figure 1:**
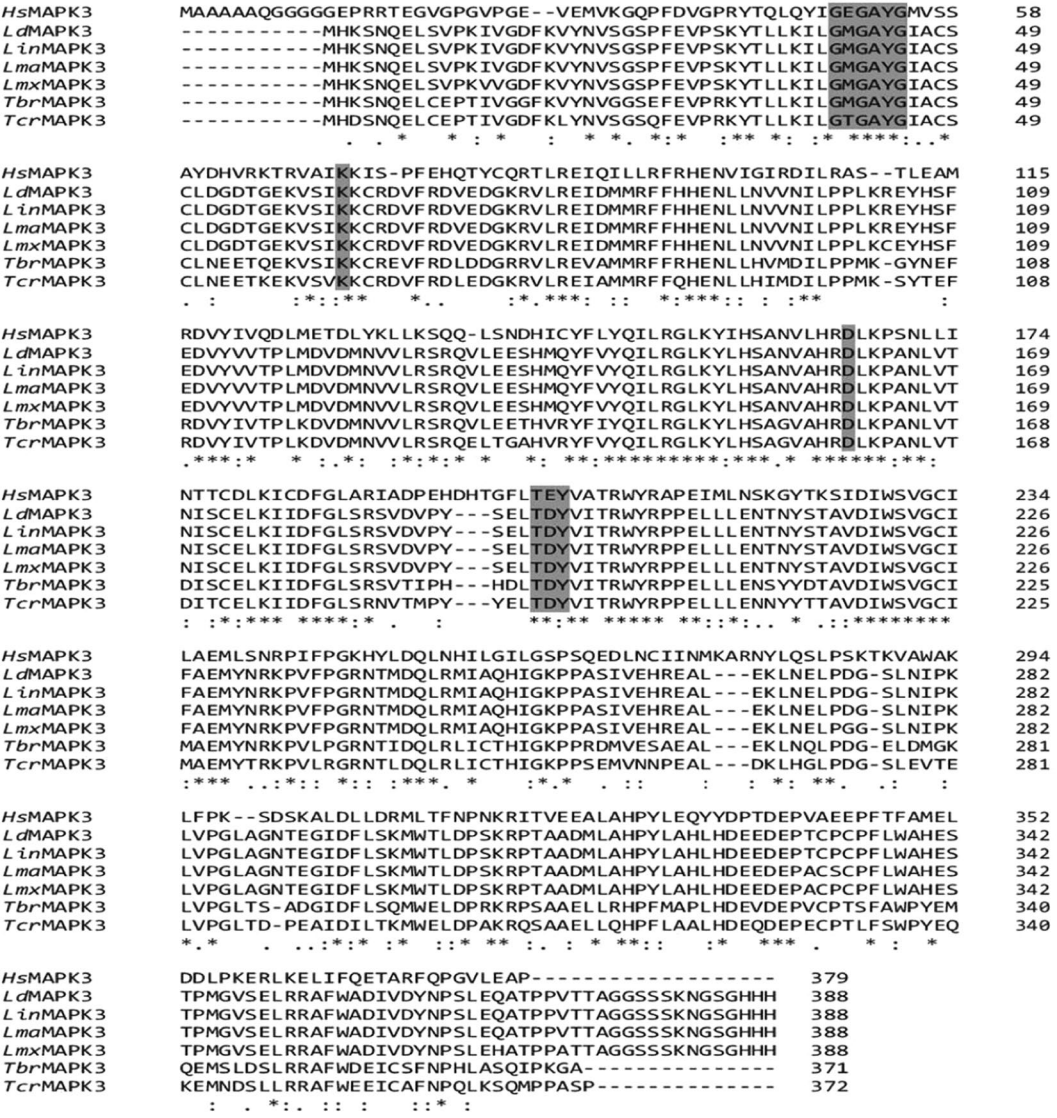
Sequence alignment of MAPK3 protein sequences from different kinetoplastids and human. The amino acid sequences are from HsMAPK3, Homo sapiens MAP kinase 3/ERK-1(BC013992.1:12-1151), *Ld*MAPK3, *Leishmania donovani* MAP kinase 3 (XM_003858842.1), *Lin*MAPK3*, Leishmania infantum* MAP kinase 3 (XM_001463665.1), *Lma*MAPK3, *Leishmania major* MAP kinase 3 (XM_001681330.1), *Lmx*MAPK3, *Leishmania mexicana mexicana* MAP kinase 3 (AJ293281.1)*, Tbr*MAPK3, *Trypanosoma brucei brucei* MAP kinase 3 (XM_842093.1)*, Tcr*MAPK3, *Trypanosoma cruzi* MAP kinase 3 (XM_802722.1). The conserved and similar amino acid residues are pointing out by asterisks and dots respectively.

*Ld*MAPK3 has an ORF of 1167 base pairs which encode for 388 amino acids with a molecular weight of 43.8 kDa. Multiple sequence analysis demonstrated the amino acid sequence similarity of *Ld*MAPK3, *Leishmania donovani* MAP kinase 3 with *Lin*MAPK3*, Leishmania infantum* MAP kinase 3 (100% identity), *Lma*MAPK3, *Leishmania major* MAP kinase 3 (99.4% identity), *Lmx*MAPK3, *Leishmania mexicana* MAP kinase 3 (98.4% identity)*, Tbr*MAPK3, *Trypanosoma brucei* MAP kinase 3 (73.3% identity) *Tcr*MAPK3, *Trypanosoma cruzi* MAP kinase 3 (71.7% identity) and *Hs*MAPK3, *Homo sapiens* MAP kinase 3/ERK-1 (40.72%)(Fig. 1). The *Ld*MAPK3 protein sequence contains the typical 12 subkinase domains of a MAP kinase protein and amino acids in these subdomains are known to be highly conserved among all kinetoplastids. Subdomain I is the ATP binding and orientation domain which forms the phosphate anchor ribbon containing the GxGxxG sequence. The subdomain II consists of an invariant conserved lysine residue (Lys62) which is responsible for catalytic activity by phosphate transfer reaction. The TXY motif which is a characteristic feature of all MAP kinases was found to be at threonine194 and tyrosine196. Kinase substrates phosphorylate MAP kinase kinase on Thr194 and Tyr196 for the activation of the enzyme.

### Cloning and expression of *Ld*MAPK3

**Figure 2:**
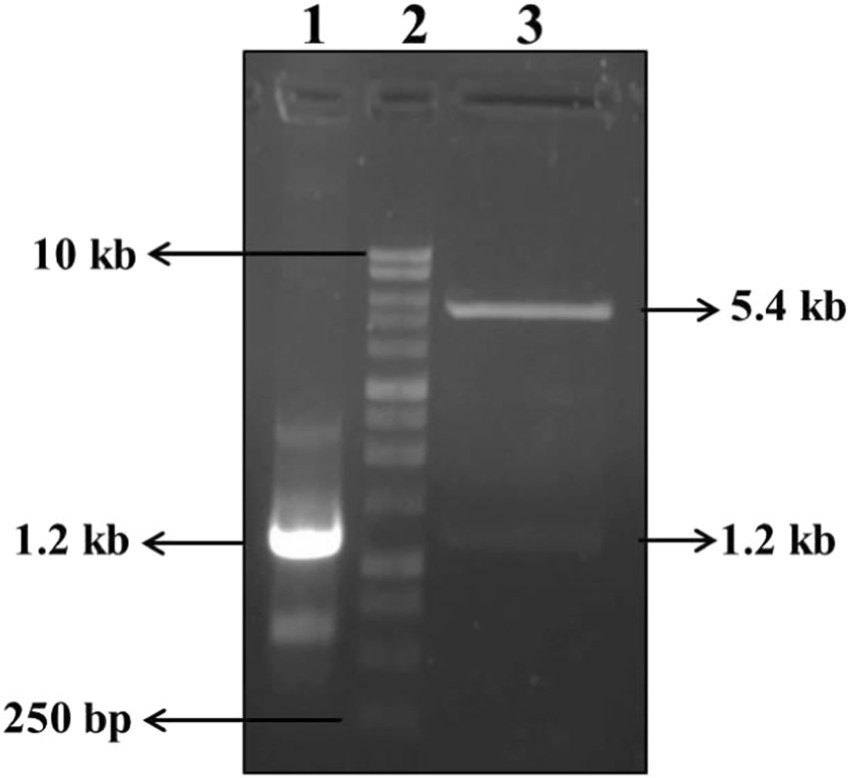
Confirmation of pET28a- *Ld*MAPK3 construct. Lane 1 showing the amplification of *Ld*MAPK3 by colony PCR method; Lane 2 is indicating 1-kb DNA ladder and Lane 3 is showing the release of insert (*Ld*MAPK3) at 1.2 kb and 5.369 kb as vector (pET 28a(+)) using restriction digestion.

**Figure 3:**
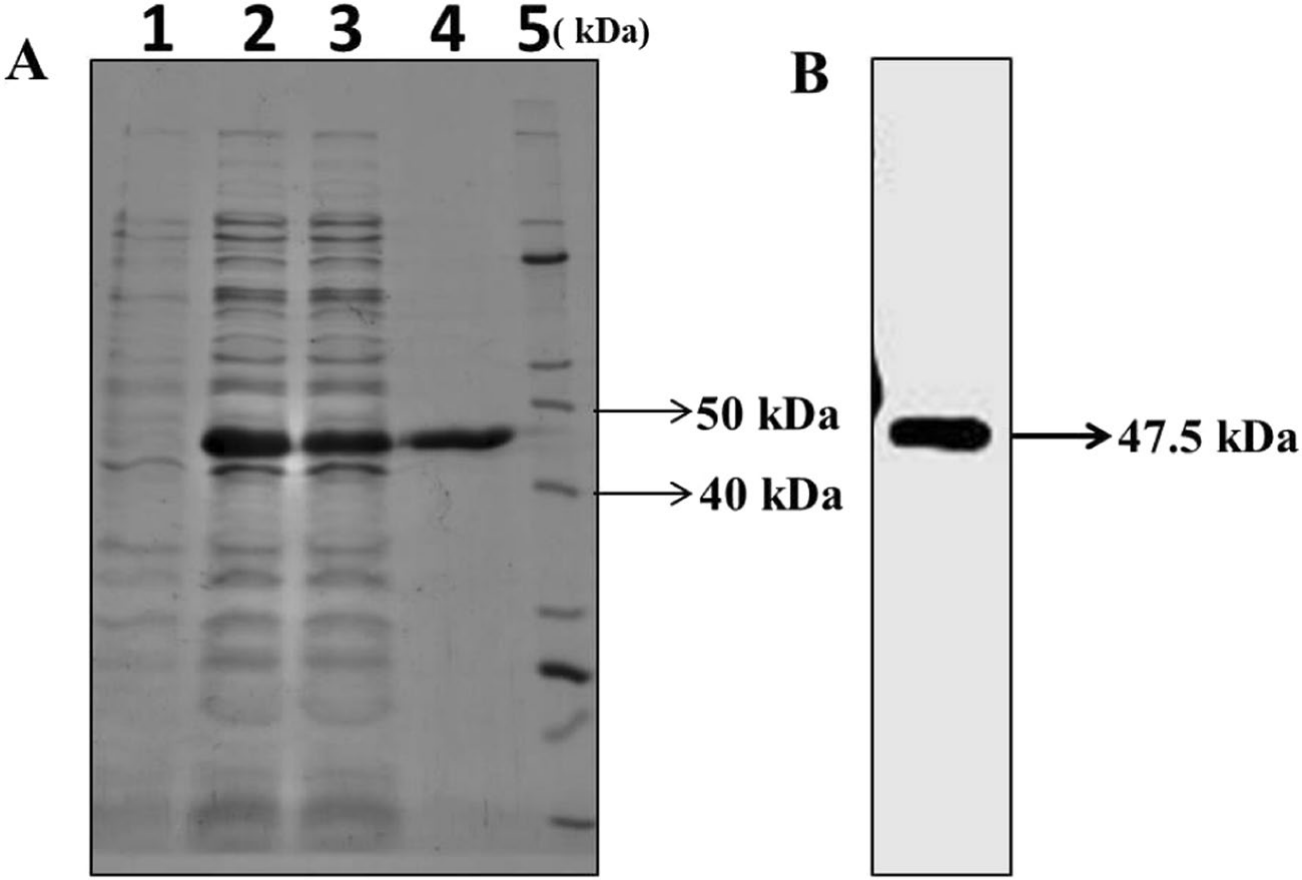
Expression and purification of *Ld*MAPK3. (**A**) Coomassie blue R250 staining: Lane 1 soluble fraction of un-induced *Ld*MAPK3 transformed in *E.coli* BL21 (DE3); Lane 2 soluble fraction of induced *Ld*MAPK3 with 0.5 mM IPTG*;* Lane 3- unbound *Ld*MAPK3 protein on Ni-NTA column; Lane 4- purified *Ld*MAPK3 eluate (~48 kDa); Lane 5- Unstained Protein Marker. (**B**) Immunoblot using His_6_ monoclonal antibody (mAb). Immunoblot developed using same eluate sample in a duplicate SDS-PAGE gel using His_6_ mAb.

The pET28a- *Ld*MAPK3 construct was confirmed by restriction digestion and colony PCR. The release of insert after restriction digestion was found to be ~1.2 kb confirming the positive construct which was later sequenced using the Sanger sequencing method (Fig. 2). The pET28a- *Ld*MAPK3 construct was transformed into *Escherichia coli* BL21 (DE3) strain for the production of enzymatically active solubilized protein. Purification of *Ld*MAPK3 was done using Ni-NTA affinity chromatography and eluted against the highest concentration of Imidazole i.e. 500 mM. Migration of *Ld*MAPK3 was analyzed using SDS-PAGE and the molecular mass of recombinant protein was found to be ~48 kDa (Fig. 3A). The yield of *Ld*MAPK3 protein was found to be around 2–4 mg/L. Immunoblot analysis was also used to analyze the recombinant *Ld*MAPK3 protein and the same was confirmed using monoclonal His_6_ anti-mouse antibody (Fig. 3B). The molecular weight of recombinant *Ld*MAPK3 protein was found in accordance with the SDS-PAGE. The orginal uncropped images of Fig. 2 and Fig. 3 are provided as supplementary information.

### Biochemical characterization of *Ld*MAPK3

**Figure 4:**
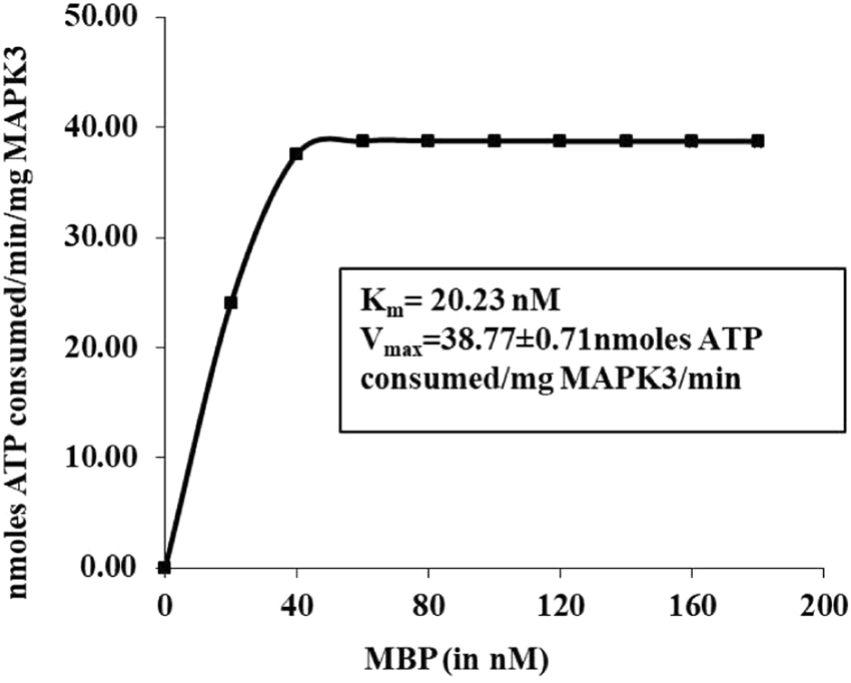
Michaelis Menton Plot: kinase activity of *Ld*MAPK3 using MBP as a substrate. Each value represents mean ± SD of three independent reactions. The K_m_ and V_max_ of *Ld*MAPK3 is clearly shown in the graph.

**Figure 5:**
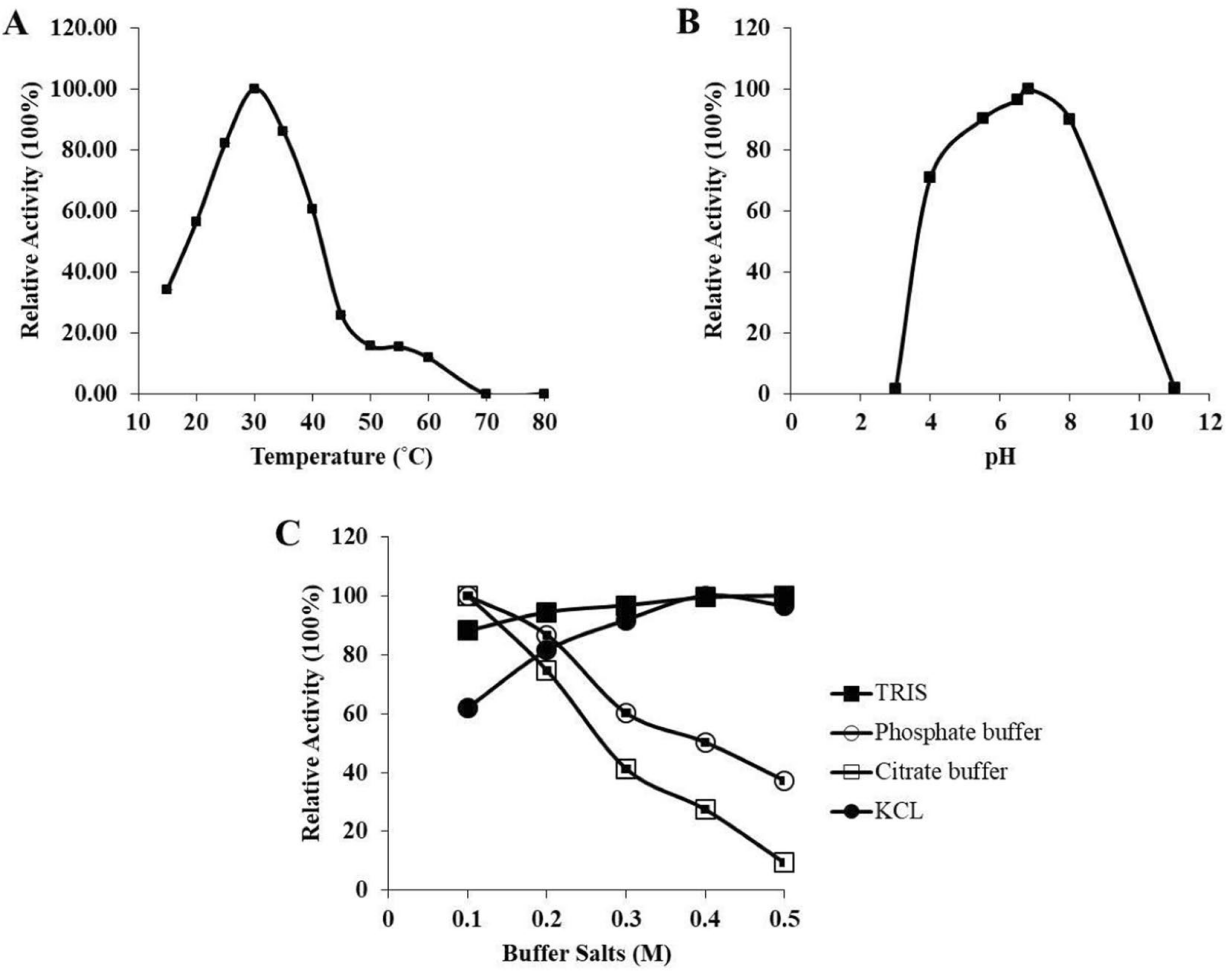
Biochemical analysis of *Ld*MAPK3. (**A**) Optimum temperature studies at different temperature conditions ranging from 10 °C–80 °C. (**B**) Optimum pH studies in mixed buffer system ranging from pH 3-11. Buffer systems with different pH ranges used in the optimum pH studies are citrate buffer (3.0–6.0), Sodium phosphate (5.8–8.0), MOPS (6.5–7.9), HEPES (6.8–8.2), Tris (8.0) and CAPS (9.7–11.1). (**C**) Stability of *Ld*MAPK3 in different buffer/salt system at different concentrations.

The kinase activity of purified recombinant *Ld*MAPK3 protein was determined using the ATP utilization method by measuring the number of ATP utilized per minute per microgram of the purified kinase. Figure 4 shows that the amount of ATP consumed per reaction is equal to the kinase activity and increases linearly with an increase in the concentration of MBP. The increment in the kinase activity was found to be concentration-dependent and observed up to 45 min. Hence, a 45 min time point was selected for inhibition studies. *Ld*MAPK3 shows a strong substrate-specific activity by giving strong signals during phosphorylation. The kinase activity follows the Michaelis-Menten equation. The K_m_ was found to be 20.23 nM with the V_max_ at 38.77 ± 0.71 nmoles ATP consumed/mg *Ld*MAPK3/min. The optimum temperature profiling followed a bell-shaped symmetrical curve giving the highest kinase activity at 35 °C (Fig. 5A). The activity decreased with increase in temperature. The optimum pH was found to be at pH 7 in different buffer systems (Fig. 5B). The stability of purified *Ld*MAPK3 protein was determined using different buffer/salt system in the kinase buffer at different concentrations. KCl at a concentration of 0.4 M was found to be the most favorable buffer condition for the kinase activity of *Ld*MAPK3 (Fig. 5C). The *Ld*MAPK3 protein was found to be quite stable for 45 days at −20 °C in glycerol.

### Inhibition studies of *Ld*MAPK3

**Figure 6:**
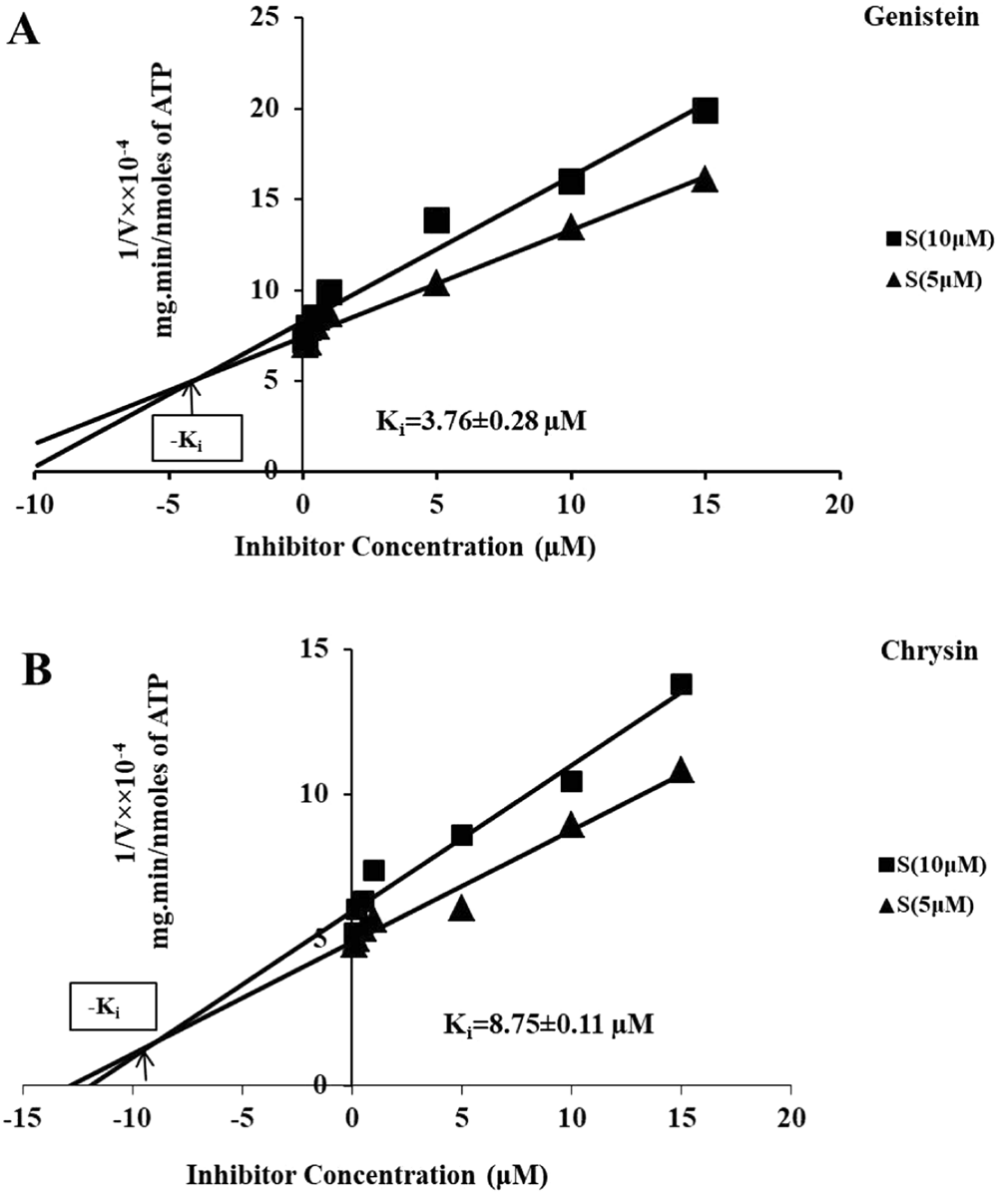
Dixon plots: Mode of inhibition by genistein and chrysin. Competitive inhibition by (**A**) genistein and (**B**) chrysin with *Ld*MAPK3 at two different substrate concentrations (5 µM and 10 µM). The intersection points of trend lines in negative x-axis indicating the K_i_ of compounds.

Natural compounds used in inhibition studies were chosen based on their binding energy values obtained from docking and virtual screening. The kinase inhibition assay was performed using two natural test compounds GEN and CHY. The *in-vitro Ld*MAPK3-mediated kinase activity was inhibited by both the test compounds individually in a dose-dependent manner. 20 µL DMSO was used as a control with the same reaction conditions. Dixon plot was plotted using two different substrate concentrations, 5 µM and 10 µM and the point of the intersection were taken as K_i_ (Fig. 6). Both the compounds exhibited a competitive mode of inhibition with respect to ATP. The K_i_ value indicates the robustness of an inhibitor i.e. higher the K_i_ value lesser is the inhibitor potency. The K_i_ for GEN and CHY phosphorylation was estimated to be 3.76 ± 0.28 µM and 8.75 ± 0.11 µM respectively. The *in-vitro* inhibition studies with *Ld*MAPK3 indicate that GEN is showing better inhibition compared to CHY. The consistency of these inhibition results was further evaluated with cell proliferation assay.

### Cytotoxicity assay

**Figure 7:**
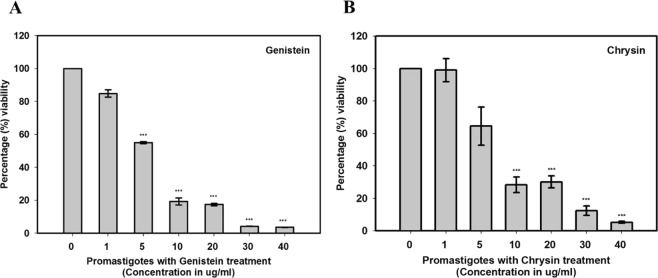
Cell proliferation assay. *In-vitro* Cytotoxic effect on *L. donovani* promastigotes treated with genistein (**A**) and chrysin (**B**). The IC_50_ values for GEN and CHY were found to be 9.9 µg/mL and 13 µg/mL respectively. Note- p < 0.001 is considered as ***.

We also investigated the impact of cytotoxicity with both the compounds on *Leishmania* promastigotes using cell proliferation assay. The viability of promastigotes showed a significant decrease after treating with 5 µg/mL concentration of these compounds. As the dosage concentration of inhibitors increased from 5–10 μg/mL, a rapid enhancement was observed in the death profile of *L. donovani* promastigotes. The inhibition studies with *Ld*MAPK3 show a higher K_i_ value for CHY but a slow death profile as compare to GEN which indicates its low anti-leishmanial property (Fig. 7). The IC_50_ value for each compound was calculated by plotting percentage cell viability vs. concentration. However, in Fig. 7B percentage viability for 10 μg/mL and 20 μg/mL of chrysin was found to be almost similar that was just an experimental coincidence. The IC_50_ values for GEN and CHY were found to be 9.9 μg/mL and 13 μg/mL respectively. The statistical analysis was performed by student’s test (unpaired) using Sigma plot 10. Though in previous experimental demonstration IC_50_ values for CHY against *Leishmania donovani, Trypanosoma brucei rhodesiense* and *Trypanosoma cruzi* were found to be 2.2 μg/mL, 5.3 μg/mL and 20.6 μg/mL respectively^42^, the variation in IC_50_ values for both the cases is due to the cellular form. The cell proliferation assay performed in our study is based on promastigotes form whereas the previously reported assay was done using amastigotes. So, with this, we can conclude that inhibition efficiency totally relies on the cellular form and thus, is stage-specific in nature.

### Infectivity assay

**Figure 8:**
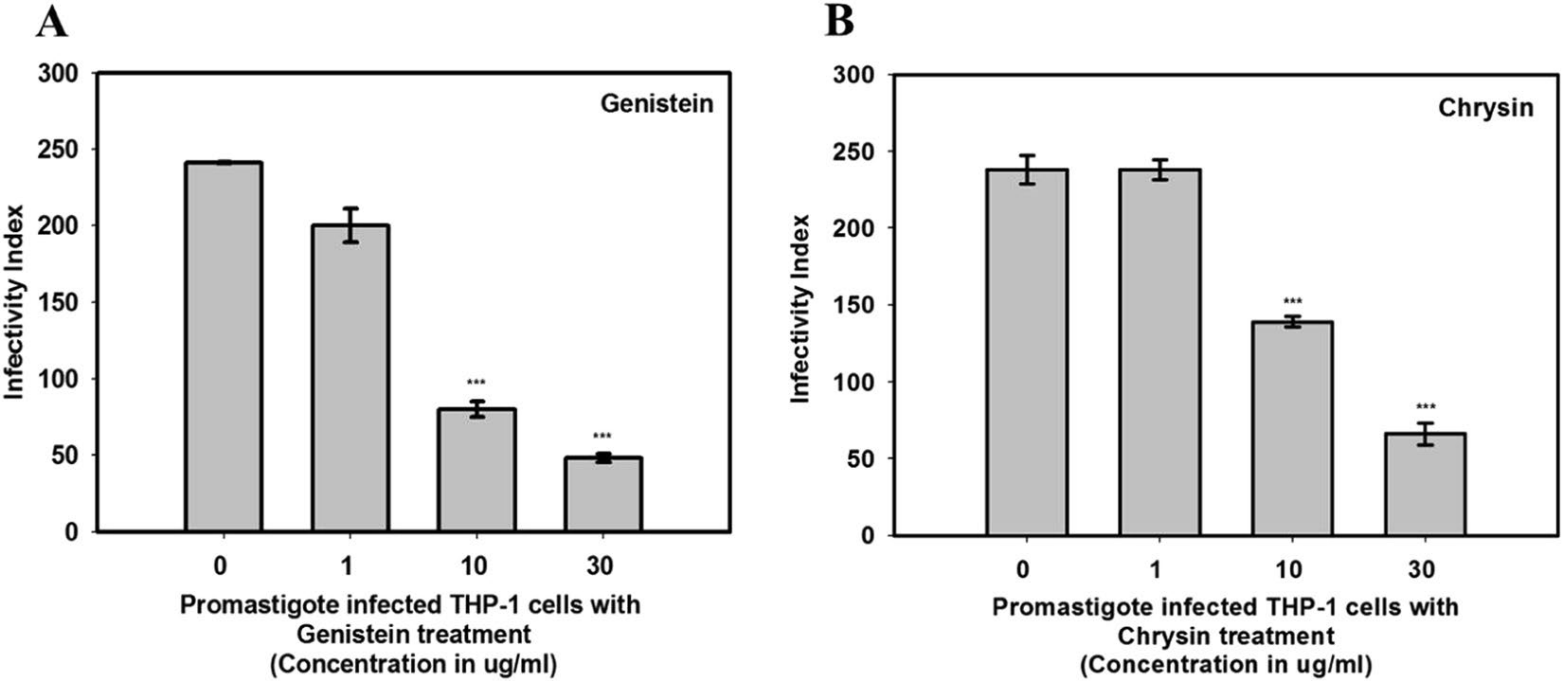
Parasite infectivity assay. *In-vitro* infectivity effect on *L. donovani* promastigotes infected with THP-1 cells after treating with genistein (**A**) and chrysin (**B**). CHY has higher infectivity index as compared to GEN. Note- p < 0.001 is considered as ***.

The process of drug discovery for anti-leishmanial compounds is mainly based on the target. However, the *in-vitro* analysis of test compounds mostly shows variations in *in-vivo* activities and therefore, this requires a more predictive *in-vitro* assay which enhances the result accuracy for the target-based screening of compounds. It has been shown that most of the anti-leishmanial drugs are based on the host cell environment^46^. PMA differentiated THP-1 cells were used to calculate the infectivity index for GEN and CHY (Fig. 8). The parasite infectivity index showed a sudden decrease in the parasite burden with the increase in the concentration of GEN. In the case of GEN, the infectivity index drastically decreased from 230 to around 75 with an increase in the concentration of GEN (1–10 µg/mL). The infectivity index showed a further reduction from 75 to 50 when THP-1 cells were treated at 30 µg/mL concentration of GEN. However, at low concentrations (1 µg/mL) CHY does not show any significant effect on parasite burden but at higher concentrations (>10 µg/mL), a gradual reduction was observed in the infectivity index. CHY showed more infectivity index as compared to GEN even at lower concentrations. Therefore, GEN showed more potency to lowers the parasite burden in treated THP-1 cells.

## Discussion

*Leishmania donovani* is the causative agent of visceral leishmaniasis which results in fatal condition unless treated. Despite the availability of contemporary medications, it still affects a very large population in undeveloped and developing countries. MAP kinases play a crucial role in the survival and proliferation of the parasite in the two distinct stress environments^33^. The MAPK3 homologue in different *Leishmania* species plays an important role in the differentiation of daughter cells by regulating the projection of new flagellum. Since MAPK3 homologue of *L. mexicana* is known to be involved in flagellar regulation and stage-differentiation, the enzyme can be an interesting target in the process of drug discovery^29^. Moreover, B-cell epitope of *L. braziliensis* MAPK3 has also been used for serodiagnosis against visceral and tegumentary leishmaniasis^47^. The recombinant MAPK3 used in serodiagnosis showed better specificity and efficacy in the immunodiagnosis of visceral leishmaniasis. Therefore, from the literature evidence, the authors conceived the idea of characterizing MAPK3 of *L. donovani* and use it as a drug target against visceral leishmaniasis. Considering the various side-effects and drug resistance of conventional anti-leishmanial chemotherapies, the study explored plant-based natural compounds for potential drug molecules^48^.

In this study, we characterized *Ld*MAPK3, a mitogen-activated protein kinase homologue of *L. donovani. Ld*MAPK3 gene of 1167 basepairs codes for MAPK3 protein containing 388 amino acids. MAPK3 showed strong similarity with other species of kinetoplastids but exhibited only 40.72% similarity with human MAPK3 gene. The decreased similarity with human MAPK3 gene served as an advantage for choosing *Ld*MAPK3 as a drug target. The *Ld*MAPK3 gene was therefore cloned into pET-28 a (+) vector and expressed in *E.coli* BL21 cells. *Ld*MAPK3 protein was consequently purified using Ni-NTA chromatography and taken up for the characterization. SDS-page analysis of *Ld*MAPK3 displayed a prominent band at 47.5 kDa and this was following the theoretically calculated molecular weight. The presence of His-tag *Ld*MAPK3 was validated by performing western blotting analysis using anti-His antibody. The activity of the protein was then checked using a non-radioactive kinase assay and K_m_ and V_max_ of *Ld*MAPK3 was calculated to be 20.23 nM and 38.77 ± 0.71 nmoles ATP consumed/mg *Ld*MAPK3/min using Michaelis-Menten kinetics. The temperature and pH characterization of *Ld*MAPK3 showed the highest activity at 35 °C and at the pH of 7. Furthermore, the buffers/salts compatibility of *Ld*MAPK3 was found to be better in KCl but the protein precipitated when stored for a short period, therefore, Tris buffer was chosen for further activity studies.

The authors wanted to utilize the drug target potentiality of *Ld*MAPK3 and therefore, natural inhibitors from databases were searched upon. Previous in-silico studies in our lab had concluded the higher binding affinity of GEN and CHY towards *Ld*MAPK3^49^. Therefore, both the compounds were individually tested against *Ld*MAPK3. The results showed low K_i_ values wherein both the inhibitors showed a competitive mode of inhibition but GEN dominated the inhibition efficiency with K_i_ value of 3.76 ± 0.28 µM over CHY (K_i_ = 8.75 ± 0.11 µM). To further validate the inhibition studies, cytotoxicity assay was performed against *L. donovani*. The MTT assay was performed on promastigote form of *L. donovani* and both the compounds showed significant cell death. The IC_50_ values for GEN and CHY were calculated to be 9.9 µg/mL and 13 µg/mL respectively. However, the IC_50_ values of evaluated compounds were higher when compared to the IC_50_ values of standard drugs like miltefosine (5.42 µg/mL)^50^ and amphotericin B (0.12 µg/mL)^51^. The standard drugs like miltefosine and amphotericin B show several side-effects and also drug resistance which makes them inappropriate for the treatment of leishmaniasis but the natural compounds used in our study are non-toxic in nature and also approved by FDA^52,53^. The parasite burden, when treated with both the compounds, can be found by infectivity index which is calculated by infectivity assay. The infectivity index in our study showed strong inhibition potency of GEN by lowering the burden of parasites on differentiated macrophages. The inhibition profiling of compounds in our performed experiments followed the same trend indicating the higher inhibition efficiency of GEN when compared to CHY. The high antioxidant capacity of GEN could also be a reason for its high anti-parasitic activity and therefore, the mechanism of activity might be the increase in ROS production inside the cells^54^. Compounds like GEN might also target other kinases in *L. donovani* which shares similar active binding sites.

This study characterized *Ld*MAPK3 which is one of the essential enzymes of *L. donovani* and also showed that GEN and CHY inhibit *Ld*MAPK3 enzyme effectively. The compounds also decreased the parasite burden on the macrophages. Although, both the compounds showed significant inhibition against *Ld*MAPK3, the potency of these compounds has to be further validated by *in-vivo* assays. The study on animal models will reflect a better picture of the application of these compounds as potential drug candidates against leishmaniasis. These natural compounds furthermore can be used in the combinatorial therapies or developed into nano-formulations for effective treatment of leishmaniasis.

## Materials and Method

### Parasite culture, cell culture, and reagents

*L. donovani* promastigotes (BHU-1081 strain) were originally obtained as a kind gift from Prof. Shyam Sundar, IMS, Banaras Hindu University, India. Briefly, promastigotes were cultured in M199 medium (Sigma) supplemented with 10% (v/v) heat-inactivated fetal bovine serum (FBS), 100 U/mL penicillin and 100 µg/mL streptomycin at 28 °C. THP-1 human monocyte cell line was procured from NCCS, Pune and grown at 37 °C with 5% CO_2_ in RPMI-1640 media supplemented with 10% (v/v) FBS, 2 mM glutamine, 100 U/ml penicillin and 100 µg/mL streptomycin. The secondary anti-mouse IgG-HRP conjugated polyclonal antibody was purchased from Abcam. The enhanced chemiluminescence (ECL) reagent substrate was purchased from BIO-RAD. Restriction enzymes, 1-kb DNA marker, stained and unstained protein markers were purchased from NEB. All the chemicals used in this study were of molecular grade and purchased from Sigma and Merck.

### Cloning of recombinant construct

The expression construct for the production of recombinant MAPK3 was created by using the gene encoding *Ld*MAPK3 from the NCBI nucleotide database with accession number (XM_003858842.1). The gene was PCR amplified from 30 ng of *L. donovani* genomic DNA- *Ld*1081-BHU (a kind gift from Dr. Shyam Sundar, IMS-BHU) using position-specific primers *5*′*-*ATA TTA GGA TCC ATG CAC AAG AGC AAC CAG*-3*′ (forward) and *5*′- GCT GGC AAG CTT CTA GTG ATG GTG GCC GCT *-3*′(reverse). The fragment was cloned into a bacterial expression vector, pET-28 a digested with *Bam*HI/*Hind*III to generate pET28a- *Ld*MAPK3. The construct was validated by colony PCR, restriction digestion and Sanger sequence analysis.

### Recombinant expression and purification of *Ld*MAPK3

*Ld*MAPK3 was expressed in *Escherichia coli* BL21 (DE3) cells. Briefly, transformed cells were grown to an optical density of 0.8 at 600 nm at 37 °C and induced with 0.5 mM Isopropyl-β-D-thio-galactopyranoside (IPTG) for 12 h at 20 °C. Cells were harvested by centrifugation at 8000 rpm for 10 min at 4 °C. Bacterial cells were resuspended in a pre-chilled buffer containing 20 mM Tris and 500 mM NaCl and 20 mM Imidazole. Samples were sonicated for a total of 50 pulses at 30 V setting on ice (5 sec on/10 sec off). Centrifugation was done at 10,000 rpm for 30 min to remove cell debris. The soluble proteins were transferred on to the column loaded with Ni-NTA agarose resin (Qiagen). The beads were washed with 10 column volumes of buffer A (20 mM Tris, 500 mM NaCl, and 20 mM Imidazole) and 5 column volumes of buffer B (20 mM Tris & 500 mM NaCl). Proteins were eluted against elution buffer containing 20 mM Tris, 500 mM NaCl, and 500 mM Imidazole. The eluate was supplemented with 15% glycerol and stored at −20 °C for further use. The purified recombinant protein was separated by using 12% SDS-PAGE and visualized by Coomassie staining using Coomassie Brilliant Blue R-250 dye.

### Western blotting of *Ld*MAPK3

Protein samples were separated on an SDS-polyacrylamide gel and transferred onto polyvinylidene difluoride (PVDF) membranes (Millipore, USA) by wet transfer method. Non-specific binding was blocked with 5% skimmed milk solution in TBST (Tris- 19 mM, NaCl-137 mM, KCl- 2.7 mM (pH 7.4) and 0.2% Tween 20) for 2–3 h at room temperature. The blots were incubated with primary His_6_ monoclonal anti-mouse antibody (1:5000 dilutions) for overnight at 4 °C on a gel rocker. Post-incubation with the primary antibody, the membrane was washed three times for 15 min each with TBST. The membrane was incubated with anti-mouse IgG-HRP-conjugated secondary antibody (1:5000 dilutions) on a rocker for 2 h at room temperature. The membrane was again washed three times for 15 min each with TBST. The blots were developed using enhanced chemiluminescence (ECL) reagent (Clarity Max Western, BIO-RAD). The image was scanned in a Gel-Doc system (Azure Biosystems).

### Kinase activity assay

The kinase activity of purified recombinant *Ld*MAPK3 protein was determined using the ATP utilization method. Kinase assay for the purified protein was performed using canonical MAPK substrate, myelin basic protein (MBP) and ATP as the phosphate donor. All the kinase reactions were carried out in 96- well solid white flat-bottom plates (Thermo Fisher Scientific). The kinase activity assay was performed using the Kinase-Glo plus luminescent assay kit (Promega). In brief, 100 μl of reaction mixture containing 50 μL of kinase buffer (50 mM Tris pH 8.0; 10 mM MgCl_2_; 2 mM MnCl_2_; 100 μM ATP), 3.125 nM of purified protein, the varying concentration of MBP and 50 μL of Glo-Max reagent was incubated for 30 min at 30 °C. The residual ATP was measured using luminometer (PerkinElmer). The kinase activity assay was standardized using different concentrations of substrate MBP (20–180 nM). K_m_ and V_max_ were calculated by using Origin Pro software 8.0. The optimum temperature for the purified protein was determined by incubating the samples in a thermocycler for 30 min in the range from 10 °C−80 °C with 12 different temperature points. The optimum pH for the purified *Ld*MAPK3 protein was determined using different buffer systems containing Tris (8.0), citrate buffer (3.0–6.0), MOPS (6.5–7.9), HEPES (6.8–8.2), Sodium phosphate (5.8–8.0) and CAPS (9.7–11.1). The ionic strength of purified *Ld*MAPK3 protein was determined using different buffer salts in the kinase buffer at different concentrations. Different buffer/salt system containing Tris (pH 8.0), KCl (pH 7.0), Citrate (pH 3.0) and Sodium phosphate (pH 5.0) with concentrations ranging from 0.1 M–0.5 M was used in the kinase assay buffer.

### Inhibition studies of *Ld*MAPK3

*In-silico* identification of natural compounds for *Ld*MAPK3 was already done in our lab (unpublished data) and top hits were used for the *in-vitro* inhibition studies. The inhibition assay was performed using two natural compounds genistein (GEN) and chrysin (CHY). The inhibition assays were carried out in 96 well-white flat bottom plate (Thermo Fisher Scientific). Each assay was performed with 100 μL of reaction mixture containing 50 μL of kinase buffer, purified *Ld*MAPK3 protein, MBP and varying concentrations of test compounds. The test compounds, GEN and CHY were dissolved in DMSO at 25 mg/mL. The reaction mixture was incubated for 30 min at 30 °C after adding an equal volume of Glo-Max reagent. The residual ATP was measured using luminometer (PerkinElmer). Data points were set for seven different concentrations of test compounds from 0.1 μM to 15 μM. For the identification of mode of inhibition by the test compounds, experimental data was tested at two different substrate concentrations (5 µM and 10 µM) for both the test compounds individually. The data sets were fitted individually for the Dixon plots to determine the inhibition patterns and K_i_ calculations.

### Cytotoxicity assay

Cytotoxicity of both the test compounds was performed using MTT cell proliferation assay. This colorimetric analysis is based on the reduction of MTT [3-(4, 5-dimethylthiazol-2-yl)-2, 5-diphenyltetrazolium bromide] dye. This tetrazolium dye was reduced by various mitochondrial enzymes into an insoluble purple color product, formazan in viable cells. Firstly, the test compounds, GEN and CHY were individually dissolved in DMSO solvent (25 mg/mL). These dissolved compounds were then serially diluted in media and added to 96-well culture plates with a concentration range of 1 – 40 μg/mL in a total volume of 100 µL. Later, the exponential growth phase promastigote cells (1 × 10^6^ cells ml^−1^) grown in M199 complete media were added to the 96 well culture plate pre-loaded with test compounds. Then, the cells were allowed to grow for 24 h at 25 °C in dark for *L. donovani* promastigotes. Lastly, MTT was added to the cells in the same 96-wells culture plates and incubated for another 4 h at 25 °C for the development of insoluble formazan. These insoluble formazan crystals which are formed after 4 h were solubilized in DMSO and absorbance was measured at 570 nm using a 96-well plate reader. The IC_50_ value indicates the concentration of test compounds that produced a 50% reduction in cell viability and calculated by plotting percentage cell viability against the concentration of compounds.

### Infectivity index

Cell environment of the primary host can mimic the biological atmosphere but shows incompatibility towards the high-throughput screening of the compounds. Alternatively, THP-1 human monocytic cell line which is derived from a leukemia patient can be used to differentiate into monocyte-macrophage cells. These differentiated macrophage-like cells can be used for the development and maintenance of *L. donovani* infections^55,56^. This infectivity assay permits the assessment of parasite viability, morphological modifications and compound toxicity that can provide a better understanding on the mode of action of test compounds against the target protein^57^. The macrophage infectivity assay was performed on THP-1 cells with the test compounds, GEN and CHY. Briefly, the human monocytic THP-1 cells were grown in 12-well culture plates with RPMI-1640 media and incubated at 37 °C for 24 h with 5% CO_2_. After 24 h, 100 ng/mL of PMA (phorbol 12 – myristate – 13 – acetate) was added to the cells to induce the differentiation of THP-1 cells into macrophages. After differentiation, cells were washed thrice using 1x PBS. After washing both the test compounds, GEN (25 mg/mL in DMSO) and CHY (25 mg/mL in DMSO) were serially diluted in media and added to the culture plate wells with a concentration range of 1 – 30 μg/mL in a total volume of 500 µL. The cells were then infected with promastigotes of *L. donovani* at the multiplication of infection (MOIs) 1:10 (macrophage: parasites) and incubated for another 24 h at 37 °C with 5% CO_2_. Lastly, amastigotes were stained using Giemsa stain and infected macrophages were counted under an inverted microscope at 60x magnification. The macrophage infectivity index was calculated using the mentioned formula:

Infectivity Index = Percentage of infected macrophages x Average number of amastigotes per infected macrophages

### Statistical analysis

In all our studies, experiments were performed individually and each data represents mean ± standard deviation which is calculated from three independent sets. For, Figs 7 and 8 statistical analysis was performed by using Sigma-Plot 10. Statistical significance was determined by student unpaired t-test and a “p” value of less than 0.001 has been considered as significant value.

## Supplementary information

Supplementary information
