# Supplementary material for: Biochemical characterization and chemical validation of Leishmania MAP Kinase-3 as a potential drug target
Source: Sci Rep. 2019 Nov 7;9:16209. doi: 10.1038/s41598-019-52774-6 (PMC6838069; doi:10.1038/s41598-019-52774-6)

Biochemical characterization and chemical validation of *Leishmania* MAP Kinase-3 as a potential drug target

Shweta Raj^1^, Gundappa Saha^3^, Santanu Sasidharan^1^, Vikash Kumar Dubey^2,^ and Prakash Saudagar^1*^

^1^ Department of Biotechnology, National Institute of Technology-Warangal, Warangal-506004. Telangana State, India.

^2^ School of Biochemical Engineering, Indian Institute of Technology-Banaras Hindu University, Uttar Pradesh-221005, India.

^3^ Department of Biosciences and Bioengineering, Indian Institute of Technology Guwahati, Guwahati, Assam, 781039, India.

*Corresponding author

Dr. Prakash Saudagar

Department of Biotechnology,

National Institute of Technology, Warangal-506004, T.S.

E-mail: [ps@nitw.ac.in](mailto:ps@nitw.ac.in)

Phone no: +91 870 2462898

1. The following figure is the original agarose gel picture of Figure 2 in the final manuscript and the cropped region is shown below.


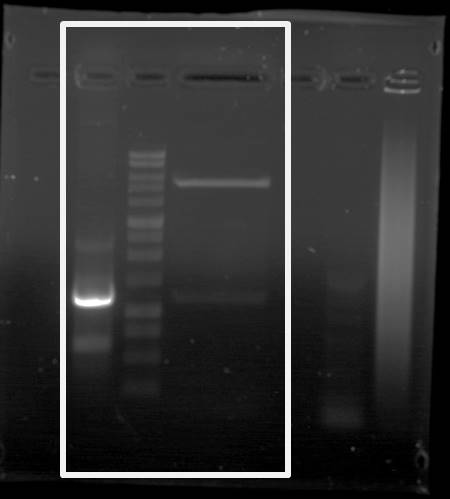


1. The following figure is the original SDS-PAGE gel picture of Figure 3A in the final manuscript and the cropped region is shown below.


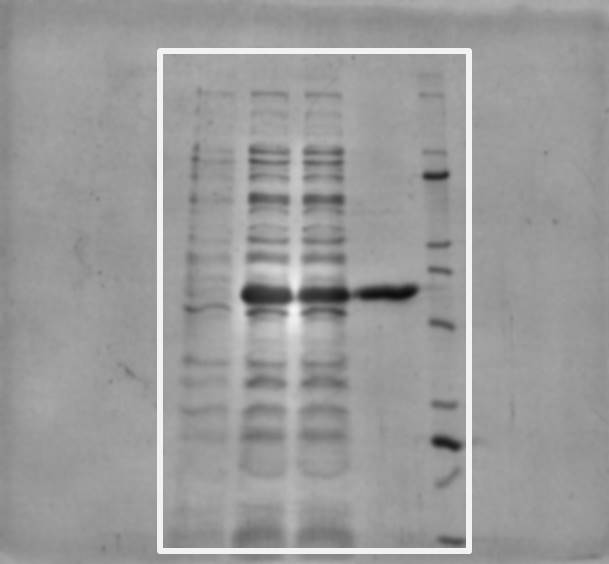


1. The order of loading of protein sample elutions on the gel was duplicated and one set of it was used for CBB staining and the other set was used for western blotting. The following figure is the original gel picture of figure 3B in the final manuscript and the cropped region is shown below.


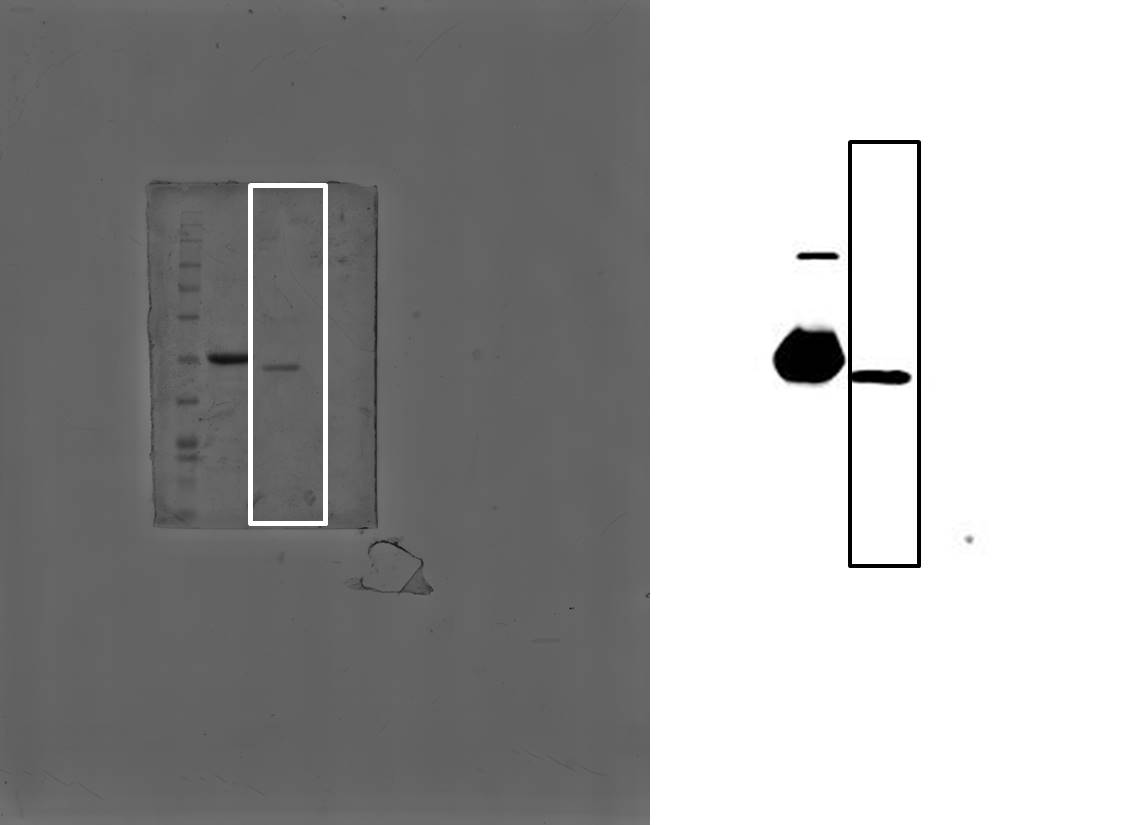

Supplement: Supplementary file 1 — Supplementary information [file 41598_2019_52774_MOESM1_ESM.docx]
